# Imatinib-Induced Interstitial Pneumonitis Successfully Switched to Nilotinib in a Patient with Prior History of Mycobacterium tuberculosis Infection

**DOI:** 10.4274/tjh.2017.0155

**Published:** 2017-12-01

**Authors:** Zhuan-Bo Luo, Ning Xu, Xiao-Ping Huang, Gui-fang Ouyang

**Affiliations:** 1 Department of Respiratory Diseases, Ningbo First Hospital, Affiliated Medical School of Ningbo University, Ningbo, China; 2 Department of Hematology, Ningbo First Hospital, Affiliated Medical School of Ningbo University, Ningbo, China

**Keywords:** Imatinib mesylate, Interstitial pneumonitis, Chronic myeloid leukemia, Nilotinib, Tuberculosis

## To The Editor,

Imatinib mesylate (IM) has been proven to be an effective treatment of chronic myeloid leukemia (CML) and this drug is well tolerated [1]. Interstitial lung disease (ILD) associated with imatinib therapy is rare. We report the case of a patient who had a prior treatment history of Mycobacterium tuberculosis infection and developed interstitial pneumonia after 10 months of imatinib for CML and who has not relapsed since the introduction of the recent tyrosine kinase inhibitor nilotinib.

**Figure 1:**
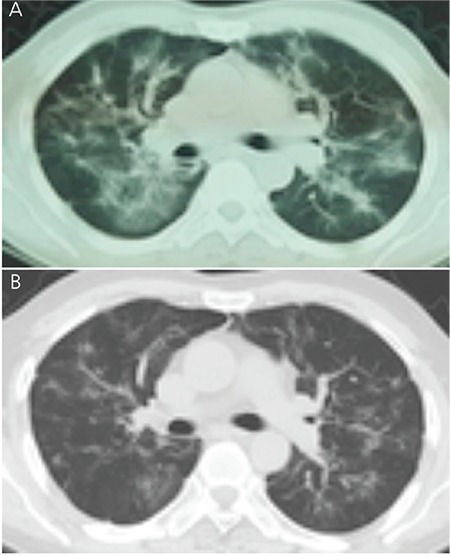
A) Chest computed tomography (CT) shows bilateral diffuse subpleural nodules, interlobular septal thickening, reticulation, and peribronchial ground glass opacities in both lungs. B) Chest CT scan 8 months after the switch to prednisone and nilotinib: lung abnormalities were decreased.

**Figure 2:**
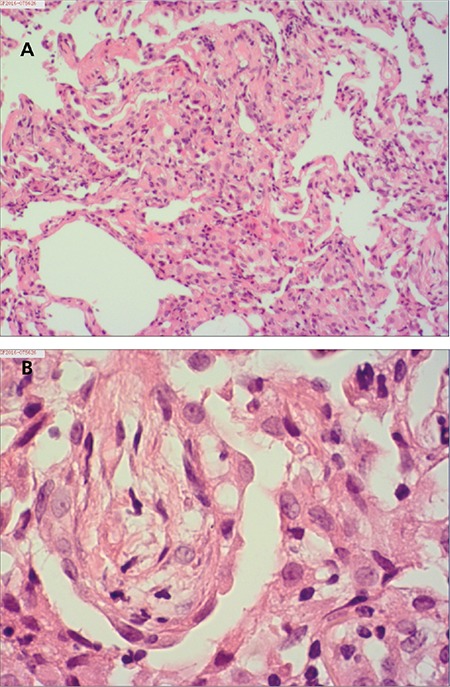
A and B) Histopathological appearance of transbronchial lung biopsy specimens. Thickened alveolar septa with modest infiltration of chronic inflammatory cells and slight interstitial fibrosis are observed. Hematoxylin and eosin stain (H&E), 100x (A); H&E stain, 400x (B).

A 48-year-old Chinese man was diagnosed with chronic-phase Philadelphia chromosome-positive CML in January 2015. His medical history was unremarkable, but he had a history of previous treatment for pulmonary tuberculosis 25 years ago. He was initially treated with IM at a dose of 400 mg daily, which was well tolerated. Complete hematological response was rapidly achieved after 2 months. Following the administration of imatinib, the patient gradually developed a dry cough and dyspnea on exertion. In November 2015, he visited the clinic because of progressing nonproductive cough. He had been treated with imatinib at a dose of 400 mg/day for 10 months. On examination, fine crackles were audible, predominantly in both posterior lower lung fields. No elevations of the acute-phase reactants were detected, and the immunoglobulin E blood level was within the normal limits. Rheumatoid factor was negative, and antinuclear antibodies were positive at 1/100 with homogeneous staining. Sputum culture was negative and no acid-fast bacilli were observed. Lung function estimation demonstrated mild impairment of gas exchange with diffusing capacity [carbon monoxide diffusion in the lung (DLCO)] of 5.61 mmol/min per kPa (51.9% predicted) and mild restrictive impairment with forced vital capacity of 3.30 L (69.1% predicted). A chest radiograph showed fibrotic scar lesions in the left upper lung field associated with the previous pulmonary tuberculosis. A computed tomography scan (Figure 1A) showed significant extension of the interstitial lung abnormalities, predominantly in the lower lobes. Bronchoscopy revealed normal airways, and histopathological analysis of the transbronchial lung biopsy demonstrated nonspecific interstitial pneumonitis, showing thickened alveolar septa with modest infiltration of chronic inflammatory cells and slight interstitial fibrosis (Figures 2A and 2B). As these findings were highly suggestive of imatinib-induced interstitial pneumonitis, this agent was discontinued and was replaced by nilotinib. At the same time, prednisone at 30 mg/day was given during the initial days, and it was slowly tapered to 10 mg/day over 2 months. Because no signs of recurrence of pulmonary tuberculosis were detected and the patient was afraid of the side effects of anti-tuberculosis drugs, we did not give anti-tuberculosis prophylaxis, but we maintained close follow-up. This resulted in a gradual improvement in his clinical condition. Partial radiological resolution was observed after 4 months and further improved at 8 months (Figure 1B). DLCO improved to 6.78 mmol/min per kPa (62.8% predicted) and forced vital capacity was 4.50 L (83.3% predicted). The switch to nilotinib at 800 mg daily was well tolerated and followed by complete cytogenetic and major molecular response sustained for 8 months.

IM is a targeted therapy that is highly active in patients with CML. It acts by inhibition of tyrosine kinase of the BCR-ABL fusion oncoprotein specific to CML. ILD is a rare adverse event associated with IM therapy. Few case series have been reported [2,3,4,5]. In the present case, the diagnosis of IM-induced ILD was made based on history, clinical symptoms, radiological findings, and pathological results. Furthermore, other etiologic factors for ILD were excluded via microbiologic and clinical studies.

Until recently, there has been a lack of data for specific risk factors for the development of IM-induced ILD. However, the incidence of the disease seems higher in patients with preexisting pulmonary diseases. The largest case series study from Japan, which analyzed 27 patients with IM-induced ILD [6], revealed that preexisting lung disease was present in more than 40% of patients with IM-induced ILD. In this case, we had found fibrotic change and pleural thickening of the left upper lung associated with prior infection of Mycobacterium tuberculosis. Thus, the case raises the possibility of the association between IM-induced ILD and airway injury related to prior infection of Mycobacterium tuberculosis. Physicians caring for a patient presenting with respiratory symptoms while on imatinib therapy should consider interstitial pneumonitis, especially in patients with previous lung or airway injuries resulting from prior infection of Mycobacterium tuberculosis.

Among the other tyrosine kinase inhibitors, similar pulmonary complications were reported with dasatinib [7] and gefitinib [8] in Japan, but not with nilotinib. In this case, 8 months after the introduction of nilotinib, interstitial pneumonitis had not recurred. Although the mechanistic basis for the absence of cross-intolerance is not fully understood, second-generation nilotinib appears to be an option in cases of ILD induced by other tyrosine kinase inhibitors.
